# Impact of Central Sensitization and Pain Catastrophizing on Patient-Reported Outcomes Differs Between Unicompartmental and Total Knee Arthroplasties

**DOI:** 10.1016/j.artd.2026.102042

**Published:** 2026-07-09

**Authors:** Kohei Kawaguchi, Ryota Yamagami, Kenichi Kono, Shuji Taketomi, Mei Lin Tay, Simon W. Young, Hiroshi Inui, Sakae Tanaka

**Affiliations:** a Department of Orthopaedic Surgery, The University of Tokyo, Tokyo, Japan; b Department of Surgery, Faculty of Medical and Health Sciences (FMHS), University of, Auckland, New Zealand; c Department of Orthopaedic Surgery, North Shore Hospital, Auckland, New Zealand; d Department of Orthopaedic Surgery, Saitama Medical Center, Saitama, Japan

**Keywords:** Central sensitization, Pain catastrophizing, Patient-reported outcomes, Total knee arthroplasty, Unicompartmental knee arthroplasty

## Abstract

**Background:**

Psychological factors have been reported to influence postoperative clinical outcomes following knee arthroplasties. The present study aimed to examine the association between preoperative central sensitization and pain catastrophizing and postoperative outcomes in patients undergoing unicompartmental knee arthroplasty (UKA) vs total knee arthroplasty (TKA).

**Methods:**

This prospective cohort study included 353 patients divided into 2 groups according to the surgical procedure: UKA (n = 72) and TKA (n = 281). The Central Sensitization Inventory (CSI) and Pain Catastrophizing Scale (PCS) questionnaires were administered preoperatively, and patient-reported outcome measures (PROMs) were evaluated according to the subscales of the Knee Injury and Osteoarthritis Outcome Score 1 year postoperatively. Preoperative CSI and PCS scores were compared between patients undergoing UKA vs TKA, and the correlations with PROMs were analyzed.

**Results:**

The mean (± standard deviation) preoperative CSI and PCS scores were 21.4 ± 10.5 and 27.3 ± 10.8 for the UKA group, and 22.6 ± 14.1 and 26.2 ± 12.9 for the TKA group; however, the differences in these scores between the groups were not statistically significant (*P* = .51 and *P* = .53, respectively). CSI and PCS scores exhibited no correlation with any postoperative subscale in PROMs (all subscales in Knee Injury and Osteoarthritis Outcome Score) in UKA. However, CSI and PCS scores exhibited negative correlations with all subscales in PROMs in TKA.

**Conclusions:**

There were no statistical differences in preoperative central sensitization and pain catastrophizing between patients who underwent UKA vs TKA. However, these psychological factors negatively affected postoperative PROMs in patients who underwent TKA, whereas, they were not found to significantly affect postoperative PROMs in the UKA group; however, this finding may reflect limited sample size rather than a true absence of effect.

## Introduction

Postoperative patient-reported outcome measures (PROMs) for total knee arthroplasty (TKA) have become popular as an alternative to subjective evaluations [1]. In fact, the results of studies using PROMs have reported that approximately 10 to 20% of patients were not satisfied after undergoing TKA [2,3]. Various risk factors for dissatisfaction with TKA have been investigated [4,5]. Preoperative psychological factors, such as central sensitization to pain, catastrophic thinking refers to a cognitive response that amplifies the perceived threat and severity of pain, depression, pessimistic thinking refers to a cognitive response that leads individuals to anticipate poorer outcomes and emphasize potential complications related to their condition or treatment, and low motivation, have been reported to influence postoperative PROMs [[6], [7], [8], [9]]. In particular, central sensitization and pain catastrophizing have been identified as preoperative psychiatric factors for postoperative dissatisfaction and painful knees after TKA [10].

Central sensitization is a condition characterized by chronic pain due to modulation of the central nervous system, reflected by sustained stimulation of peripheral nociceptors, causing inactivation of the descending inhibitory system. Typical symptoms of central sensitization include hyperalgesia, allodynia, and persistent pain [11]. It has been reported that 20%–40% of patients with advanced knee osteoarthritis already experience central sensitization to pain [12] and central sensitization has recently been the focus of interest as a potential cause of persistent pain and discomfort after TKA [13].

Pain catastrophizing is defined as an exaggerated negative reaction to real and/or imaged pain [14]. Pain catastrophizing is a process in which the experience of pain is viewed pessimistically. When catastrophic thinking is strong, there is excessive fear of pain, which leads to a negative cycle of reduced activity due to behavioral avoidance, resulting in a state of chronic pain [15]. Several studies have reported an association between the postoperative clinical outcomes of TKA and preoperative pain catastrophization [[16], [17], [18], [19]]. Preoperative pain catastrophizing has been found to be associated with poor pain scores [[20], [21], [22]], poor activity, and prolonged hospital stay [23].

Unicompartmental knee arthroplasty (UKA) is performed for single-compartment knee disorders, whereas TKA is typically performed for knee disorders involving 2 or more compartments. In comparisons of postoperative PROMs between the 2 procedures, UKA has been reported to yield superior outcomes than those for TKA [24]. However, it remains unclear whether preoperative central sensitization and pain catastrophizing differ between the 2 procedures, which have different preoperative ranges of motion and degrees of osteoarthritis. Additionally, there have been no studies comparing the effects of these 2 psychological factors on postoperative PROMs between TKA and UKA. The aims of the present study, therefore, were to compare preoperative central sensitization and pain catastrophizing between patients undergoing UKA vs TKA, and to examine the association of the 2 psychological factors with postoperative PROMs in UKA compared with TKA.

## Material and methods

**Figure 1:**
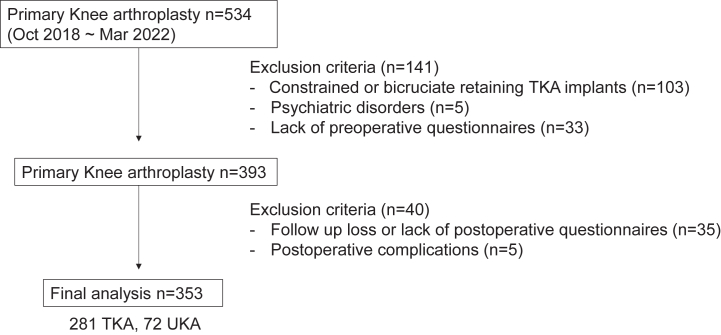
Flowchart of included and excluded patients in this prospective cohort study.

This was a cohort study of patients scheduled for elective primary knee arthroplasty and analyzed retrospectively when data were completed. Patients were eligible for inclusion in this study if they underwent primary UKA or TKA, had complete preoperative psychological assessments, and ≥1 year(s) of follow-up. In total, data from 353 knee arthroplasties (281 TKA, 72 UKA) from consecutive 534 primary knee arthroplasties performed at the single institution between October 2018 and March 2022 were included. In total, 103 knees, including constrained, postligament reconstruction, and bicruciate-retaining TKA, were excluded. Patients with psychiatric disorders (schizophrenia) who required psychiatric intervention during their hospitalization were excluded (n = 5), those without psychological assessments (n = 33), those with postoperative complications (n = 5), and those lost to follow-up (n = 35) were also excluded (Fig. 1), and bilateral cases were assessed separately. Preoperative psychological measurements and associations between preoperative psychological measures and postoperative PROMs were compared for UKA and TKA. The institutional review board approved this study (No.2674). The patients and their families were notified that their anonymized case data would be published, and all patients provided informed written consent. This retrospective observational study was reported in accordance with the STROBE statement.

### Surgical procedures

All UKA patients received the same mobile-bearing medial UKA implant (Oxford Medial Partial Knee; Zimmer Biomet, Warsaw, NJ, USA) for medial knee osteoarthritis (n = 52) or osteonecrosis of the femoral condyle (n = 20). No lateral UKA was observed in this study. Surgical procedures and postoperative clinical protocols were standardized. The approach used a microplasty system and image-free navigation system (Precision N, Stryker Orthopedics, Mahwah, NJ, USA). For TKA, the implants used were the Journey II bicruciate substituting system (Smith & Nephew, Memphis, TN, USA; n = 224), a posterior-stabilized, fixed-bearing, dual-radius design; the GMK Sphere (Medacta, Castel San Pietro, Switzerland; n = 34), a cruciate-substituting, fixed-bearing, single-radius design; and the Vanguard ROCC (Zimmer Biomet; n = 23), a cruciate-substituting, mobile-bearing, multi-radius design. These implants were used in patients with osteoarthritis or moderate osteonecrosis. The choice of implant was based on patient knee deformity and surgeon preference. Surgical methods were standardized across the 3 implants, including coronal alignment strategy, surgical approach, cemented fixation, and layered closure without a drainage tube. The medial parapatellar approach was performed using an image-free navigation system (Precision N) to achieve coronal mechanical alignment in all cases. Full weight-bearing and rehabilitation were started the day after surgery for both procedures. All UKA and TKA procedures were performed by 4 experienced senior surgeons. All patients followed the same rehabilitation protocol in both UKA and TKA. On postoperative day 1, range of motion exercises and assisted walking with a walker were initiated, followed by progression to walking using a crutch. Patients were typically discharged 2 weeks after surgery and completed their rehabilitation with physiotherapists on an outpatient basis.

### Preoperative psychological examination

All patients were assessed for preoperative psychological conditions when they were admitted to the hospital 1 day before surgery. Central sensitization was assessed using the Central Sensitization Inventory (CSI) questionnaire, which is a validated self-reported inventory for evaluation of patients with central sensitization [25,26]. The inventory uses a 25-item questionnaire to evaluate somatic and emotional symptoms, and pain-sensitivity experiences. Each item is graded on a 5-point Likert scale ranging from 0 to 4 points (0 = never; 1 = rarely; 2 = sometimes; 3 = often; and 4 = always). The CSI score ranges from 0 to 100, with 0 representing the best score and 100 representing the worst score [26]. A CSI score threshold of 40 was used to categorize patients into high and low CSI groups; this threshold has been widely to detect patients with the central sensitization in previous studies [[27], [28], [29]]. Pain catastrophizing was evaluated using the Pain Catastrophizing Scale (PCS), which includes 3 different elements: helplessness, rumor, and enhancement [14,30]. The PCS consists of 13 items, each of which is graded on a 5-point Likert scale ranging from 0 to 4 points (0 = not at all; 1 = slight; 2 = moderate; 3 = great; and 4 = all the time). The PCS score ranges from 0 to 52, with 0 representing the best score and 52 representing the worst. A threshold of 30 points was used to identify the high PCS score group based on previous studies regarding knee osteoarthritis [10,22].

### Postoperative clinical outcomes

Postoperative clinical outcomes were evaluated in accordance with the 2022 Knee Society Scoring System (KSS) [31] and the Knee Injury and Osteoarthritis Outcome Score (KOOS) [32] at the 1-year postoperative clinic. Four separate categories in the KSS (objective knee score, patient satisfaction score, patient expectation score, and functional knee score) and 5 separate categories in the KOOS (pain, symptoms, activities of daily living, sports, and quality of life) were evaluated. Knee range of motion was also measured preoperatively using a standard clinical goniometer when the patients were admitted to the hospital 1 day before surgery.

### Statistical analysis

Statistical analyses were performed using SPSS version 25.0 (IBM Corp., Armonk, NY, USA). To compare TKA with UKA, comparisons of means were performed using either the Mann–Whitney U test or the independent *t*-test, depending on the normality of continuous variables was assessed using the Shapiro–Wilk test. Correlations between psychological measures and postoperative PROMs were analyzed using Pearson’s correlation coefficients. To investigate the impact of psychological measurements on PROMs, multivariable regression analyses were conducted, considering factors such as age at surgery, time interval since surgery, body mass index (BMI), sex, preoperative knee flexion angle and preoperative knee extension angle, and the psychological factor (CSI or PCS) in TKA and UKA. All statistical tests were 2-tailed, and differences with *P* < .05 were considered to be significant. Before this investigation, a power analysis was performed using G∗Power version 3.1.9.4 (Heinrich-Heine University, Düsseldorf, Germany). Using an effect size of 0.5, this required a sample size >64 in each group (1 – β = 0.80, α = 0.05).

## Results

**Figure 2:**
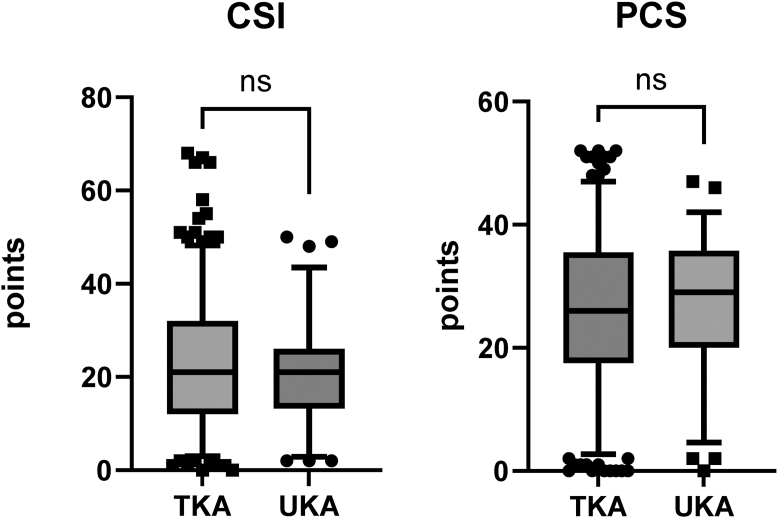
Comparison between total knee arthroplasty and unicompartmental knee arthroplasty in Central Sensitization Inventory and Pain Catastrophizing Scale. CSI, central sensitization inventory, ns, not significantly different, the box represents the interquartile range (IQR) and median of the data.

Preoperative demographic data are summarized in Table 1. There were no differences in age, BMI, or laterality between the UKA and TKA groups; however, there were more males and better preoperative knee range of motion in the UKA group than those in the TKA group. The mean (± standard deviation) preoperative CSI and PCS scores were 22.6 ± 14.1 and 26.2 ± 12.9 in the TKA group, and 21.4 ± 10.5 and 27.3 ± 10.8 in the UKA group, respectively; however, there were no statistical differences between the groups (Fig. 2). The proportion of patients in the high CSI group was similar, with 12.8% (36/281 knees) in the TKA group and 5.6% (4/72 knees) in the UKA group (*P* = .09). The proportion of patients in the high PCS group was also similar, with 39.5% (111/281 knees) in the TKA group and 48.6% (35/72 knees) in the UKA group (*P* = .16).

**Table 1:** Preoperative patient demographics in total knee arthroplasty (TKA) and unicompartmental knee arthroplasty (UKA).

Items	TKA	UKA	*P* value
Knees (patients)	281 (240)	72 (68)	
Age (years old)	73.3 ± 8.6	72.8 ± 10.2	.69
Height (m)	1.53 ± 0.08	1.57 ± 0.07	<.01^a^
Weight (kg)	61.1 ± 12.1	61.9 ± 12.8	.63
BMI (kg/m2)	25.7 ± 4.2	25.0 ± 4.0	.19
Women: men	231 : 50	48 : 42	<.01^a^
Right: left	144 : 137	37 : 35	.98
Knee extension angle (°)	7.8 ± 7.6	3.4 ± 3.4	<.01^a^
Knee flexion angle (°)	119.0 ± 17.5	131.3 ± 8.4	<.01^a^

a *P* < .05.

**Figure 3:**
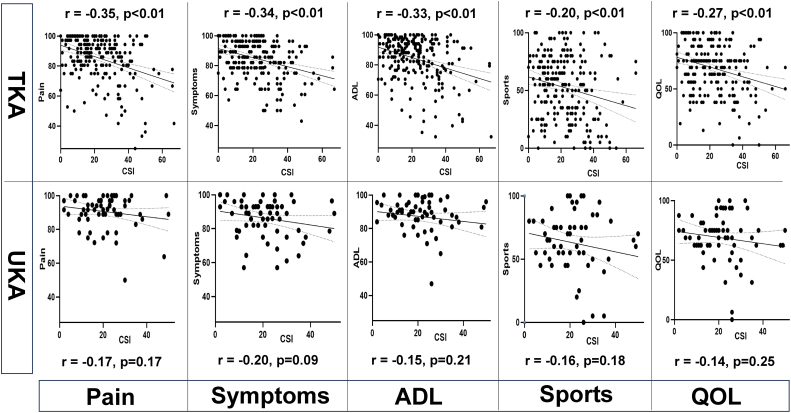
Correlations between Central Sensitization Inventory (CSI) scores and KOOS subscale scores in TKA and UKA groups. CSI scores exhibited no correlation with any postoperative subscales in KOOS in the UKA group, whereas, CSI scores exhibited negative correlations with all subscales in KOOS in the TKA group. ADL, activities of daily living; QOL, quality of life; CSI, central sensitization inventory; the Pearson correlation coefficients (r) and corresponding *P* values between preoperative CSI scores and each subscale of the Knee Injury and Osteoarthritis Outcome Score (KOOS).

**Figure 4:**
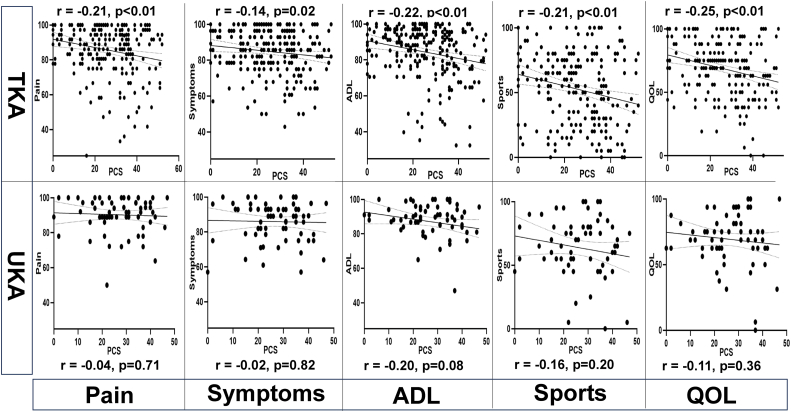
Correlations between Pain Catastrophizing Scale (PCS) scores and KOOS subscale scores in TKA and UKA groups. PCS scores exhibited no correlation with any postoperative subscales in KOOS in the UKA group, whereas, PCS scores exhibited negative correlations with all subscales in KOOS in the TKA group. ADL, activities of daily living; QOL, quality of life; PCS, pain catastrophizing scale; the Pearson correlation coefficients (r) and corresponding *P* values between preoperative PCS scores and each subscale of the Knee Injury and Osteoarthritis Outcome Score (KOOS).

Comparing postoperative PROMs between the UKA and TKA groups, UKA had better outcomes in terms of the Pain and Sports score in the KOOS; however, TKA had better satisfaction scores and objective knee score in the KSS (Table 2). Regarding the relationship between psychological factors and PROMs, preoperative CSI and PCS scores were negatively correlated with all postoperative PROMs for TKA; however, preoperative CSI and PCS scores were not correlated with all postoperative PROMs for UKA (Tables 3 and 4, Figs. 3 and 4). Multivariable regression analysis, including age, BMI, sex, knee flexion and extension angle, and psychological factors (CSI or PCS), showed CSI and PCS were influential factors for all postoperative PROMs for TKA, while CSI and PCS were not significant influential factors in UKA (Tables 5 and 6). The high CSI group exhibited inferior PROMs in TKA; however, there was no difference between the high CSI and control groups in UKA (Table 7). The high PCS group exhibited inferior PROMs, except for the KOOS symptom score in TKA; however, there was no difference between the high PCS group and the control group, except for functional score in UKA (Table 8).

**Table 2:** Postoperative patient-reported outcome measures (PROMs) and knee range of motion in total knee arthroplasty (TKA) and unicompartmental knee arthroplasty (UKA).

Items	TKA	UKA	*P* value
KOOS			
Pain	85.9 ± 14.6	90.3 ± 9.9	.02^a^
Symptoms	84.9 ± 12.2	86.0 ± 11.0	.52
ADL	84.4 ± 14.1	87.0 ± 10.5	.15
Sports	52.7 ± 27.3	63.5 ± 24.1	<.01^a^
QOL	68.8 ± 21.7	69.2 ± 19.7	.91
KSS			
Objective knee score	19.9 ± 3.6	18.5 ± 6.3	.08
Patient satisfaction score	28.8 ± 8.1	26.1 ± 10.4	.04^a^
Patient expectation score	10.1 ± 2.9	10.8 ± 2.9	.06
Functional knee score	71.9 ± 18.7	70.2 ± 20.7	.49
Knee range of motion (°)	122.0 ± 14.8	130.5 ± 9.2	<.01^a^

Average ±standard deviation.

KSS, 2022 Knee Society Scoring System; ADL, activities of daily living; QOL, quality of life.

a *P* < .05.

**Table 3:** Correlation of preoperative Central Sensitization Inventory (CSI) and postoperative patient-reported outcome measures (PROMs) in total knee arthroplasty (TKA) and unicompartmental knee arthroplasty (UKA).

PROMs	TKA				UKA			
	Correlation coefficient	95% CI		*P* value	Correlation coefficient	95% CI		*P* value
KOOS								
Pain	−0.35	−0.45	−0.23	<.01^a^	−0.17	−0.39	0.07	0.17
Symptoms	−0.34	−0.44	−0.22	<.01^a^	−0.20	−0.42	0.04	0.09
ADL	−0.33	−0.43	−0.21	<.01^a^	−0.15	−0.38	0.08	0.21
Sports	−0.20	−0.32	−0.07	<.01^a^	−0.16	−0.39	0.07	0.18
QOL	−0.27	−0.38	−0.14	<.01^a^	−0.14	−0.37	0.10	0.25
KSS								
Objective knee score	−0.27	−0.38	−0.14	<.01^a^	−0.10	−0.33	0.13	0.38
Patient satisfaction score	−0.29	−0.40	−0.16	<.01^a^	−0.11	−0.33	0.13	0.35
Patient expectation score	−0.16	−0.28	−0.03	.01^a^	−0.01	−0.24	0.23	0.95
Functional knee score	−0.30	−0.41	−0.17	<.01^a^	−0.12	−0.35	0.12	0.34

PROMs, patient-reported outcomes; KSS, 2022 Knee Society Scoring System; ADL, activities of daily living; QOL, quality of life; 95% CI, 95% confidence interval.

a *P* < .05.

**Table 4:** Correlation of preoperative Pain Catastrophizing Scale (PCS) and postoperative patient-reported outcome measures (PROMs) in total knee arthroplasty (TKA) and unicompartmental knee arthroplasty (UKA).

Items	TKA				UKA			
	Correlation coefficient	95% CI		*P* value	Correlation coefficient	95% CI		*P* value
KOOS								
Pain	−0.21	−0.32	−0.08	<.01^a^	−0.04	−0.28	0.19	0.71
Symptoms	−0.14	−0.26	−0.01	.02^a^	−0.02	−0.26	0.21	0.82
ADL	−0.22	−0.33	−0.10	<.01^a^	−0.20	−0.42	0.03	0.08
Sports	−0.21	−0.32	−0.10	<.01^a^	−0.16	−0.38	0.08	0.20
QOL	−0.25	−0.36	−0.12	<.01^a^	−0.11	−0.34	0.13	0.36
KSS								
Objective knee score	−0.20	−0.32	−0.07	<.01^a^	−0.17	−0.39	0.06	0.14
Patient satisfaction score	−0.23	−0.34	−0.10	<.01^a^	−0.19	−0.41	0.04	0.10
Patient expectation score	−0.18	−0.30	−0.05	<.01^a^	−0.15	−0.38	0.08	0.19
Functional knee score	−0.21	−0.33	−0.08	<.01^a^	−0.23	−0.45	0.03	0.05

KSS, 2022 Knee Society Scoring System; ADL, activities of daily living; QOL, quality of life; 95% CI, 95% confidence interval.

a *P* < .05.

**Table 5:** Multivariable analyses of patient-reported outcomes with CSI scores in TKA and UKA.

TKA					
PROMs	Influential factor	β value	95% CI		*P* value
KOOS Pain	Age	0.376	0.198	0.553	<.01^a^
	BMI	7.379	2.405	12.353	<.01^a^
	Woman/man	0.725	0.325	1.125	<.01^a^
	Knee flexion angle	0.304	0.199	0.410	<.01^a^
	CSI	−0.357	−0.500	−0.214	<.01^a^
KOOS Symptoms	Age	0.571	0.423	0.720	<.01^a^
	BMI	5.693	1.529	9.858	<.01^a^
	Woman/man	0.743	0.409	1.078	<.01^a^
	Knee flexion angle	0.179	0.090	0.267	<.01^a^
	CSI	−0.261	−0.380	−0.141	<.01^a^
KOOS ADL	CSI	−0.368	−0.498	−0.237	<.01^a^
KOOS Sports	BMI	13.474	4.405	22.544	<.01^a^
	CSI	−0.455	−0.718	−0.192	<.01^a^
KOOS QOL	CSI	−0.473	−0.682	−0.265	<.01^a^
KSS objective knee score	CSI	−0.071	−0.106	−0.036	<.01^a^
KSS patient satisfaction score	CSI	−0.177	−0.254	−0.099	<.01^a^
KSS patient expectation score	CSI	−0.033	−0.062	−0.005	.02^a^
KSS functional knee score	CSI	−0.406	−0.581	−0.230	<.01^a^

UKA					
PROMs	Influential factor	β value	95% CI		*P* value
KOOS Pain	Age	0.404	0.197	0.611	<0.01^a^
	Woman/man	1.040	0.532	1.548	<0.01^a^
	Knee flexion angle	0.267	0.114	0.419	<0.01^a^
	CSI	−0.054	−0.292	0.183	0.65
KOOS Symptoms	CSI	−0.110	−0.386	0.167	0.43
KOOS ADL	Age	−0.467	−0.761	−0.173	<0.01^a^
	CSI	−0.105	−0.354	0.143	0.40
KOOS Sports	Age	−0.774	−1.475	−0.073	0.03^a^
	CSI	−0.257	−0.849	0.335	0.39
KOOS QOL	CSI	−0.132	−0.647	0.384	0.61
KSS objective knee score	CSI	−0.031	−0.195	0.134	0.71
KSS patient satisfaction score	CSI	−0.042	−0.314	0.231	0.76
KSS patient expectation score	CSI	−0.021	−0.093	0.051	0.55
KSS functional knee score	Age	−0.697	−1.257	−0.137	0.01^a^
	CSI	−0.232	−0.750	0.285	0.37

CSI, central sensitization inventory; KSS, 2022 Knee Society Scoring System; ADL, activities of daily living; QOL, quality of life; PROMs, patient-reported outcome measures; 95% CI, 95% confidence interval.

a *P* < .05.

**Table 6:** Multivariable analyses of patient-reported outcomes with pain catastrophizing scale (PCS) in TKA and UKA.

TKA					
PROMs	Influential factor	β value	95% CI		*P* value
KOOS Pain	PCS	−0.253	−0.400	−0.105	<.01^a^
KOOS Symptoms	Age	0.266	0.077	0.456	<.01^a^
	PCS	−0.151	−0.273	−0.028	.01^a^
KOOS ADL	BMI	4.842	0.044	9.641	.04^a^
	PCS	−0.235	−0.377	−0.093	<.01^a^
KOOS Sports	BMI	14.021	4.993	23.049	<.01^a^
	PCS	−0.457	−0.725	−0.189	<.01^a^
KOOS QOL	PCS	−0.433	−0.647	−0.218	<.01^a^
KSS objective knee score	PCS	−0.058	−0.096	−0.021	<.01^a^
KSS patient satisfaction score	PCS	−0.146	−0.229	−0.064	<.01^a^
KSS patient expectation score	PCS	−0.045	−0.075	−0.015	<.01^a^
KSS functional knee score	BMI	6.383	0.129	12.637	.04^a^
	Woman/man	−0.623	−1.207	−0.039	.03^a^
	PCS	−0.317	−0.511	−0.124	<.01^a^

UKA					
PROMs	Influential factor	β value	95% CI		*P* value
KOOS Pain	PCS	−0.053	−0.257	0.151	0.61
KOOS Symptoms	PCS	−0.018	−0.277	0.240	0.89
KOOS ADL	Age	−0.438	−0.726	−0.150	<0.01^a^
	PCS	−0.141	−0.366	0.085	0.22
KOOS Sports	Age	−0.731	−1.422	−0.040	0.03^a^
	PCS	−0.240	−0.782	0.302	0.38
KOOS QOL	PCS	−0.141	−0.611	0.330	0.55
KSS objective knee score	PCS	−0.107	−0.264	0.049	0.17
KSS patient satisfaction score	PCS	−0.172	−0.431	0.087	0.19
KSS patient expectation score	Woman/man	−0.216	−0.425	−0.007	0.04^a^
	PCS	−0.049	−0.123	0.024	0.19
KSS functional knee score	Age	−0.610	−1.154	−0.066	0.02^a^
	PCS	−0.434	−0.961	0.092	0.10

KSS, 2022 Knee Society Scoring System; ADL, activities of daily living; QOL, quality of life; PROMs, patient-reported outcome measures.

a *P* < .05.

**Table 7:** Comparison of PROMs between high and low central sensitization inventory (CSI).

TKA				UKA		
PROMs	CSI ≥40	CSI <40	*P* value	CSI ≥40	CSI <40	*P* value
KOOS						
Pain	73.8 ± 22.0	87.2 ± 12.9	<.01^a^	84.3 ± 18.5	90.8 ± 9.3	.25
Symptoms	78.3 ± 13.6	85.7 ± 11.9	<.01^a^	88.1 ± 9.0	86.2 ± 11.2	.76
ADL	75.0 ± 19.6	85.5 ± 13.0	<.01^a^	89.7 ± 14.1	87.0 ± 10.8	.67
Sports	41.3 ± 33.4	54.2 ± 26.3	.02^a^	65.0 ± 5.0	62.9 ± 24.8	.88
QOL	56.3 ± 23.0	70.2 ± 21.1	<.01^a^	66.7 ± 7.2	69.0 ± 20.3	.84
KSS						
Objective knee score	17.2 ± 4.9	20.1 ± 3.4	<.01^a^	19.6 ± 1.2	18.5 ± 6.5	.76
Patient satisfaction score	23.7 ± 9.2	29.4 ± 7.8	<.01^a^	30.0 ± 5.3	26.0 ± 10.8	.52
Patient expectation score	9.0 ± 3.0	10.1 ± 2.9	.05	10.3 ± 1.2	10.9 ± 2.9	.74
Functional knee score	60.2 ± 20.9	73.2 ± 18.1	<.01^a^	74.6 ± 13.6	69.6 ± 21.2	.68

PROMs, patient-reported outcome measures; CSI, central sensitization inventory; KSS, 2022 Knee Society Scoring System; ADL, activities of daily living; QOL, quality of life.

a *P* < .05.

**Table 8:** Comparison of PROMs between high and low Pain Catastrophizing Scale (PCS).

TKA				UKA		
PROMs	PCS ≥30	PCS <30	*P* value	PCS ≥30	PCS <30	*P* value
KOOS						
Pain	82.8 ± 16.7	87.8 ± 12.9	<.01^a^	90.4 ± 8.6	90.1 ± 10.8	.91
Symptoms	83.2 ± 13.2	86.1 ± 11.5	.06	85.5 ± 10.3	86.4 ± 11.8	.72
ADL	81.0 ± 15.6	86.7 ± 12.8	<.01^a^	84.4 ± 12.6	89.5 ± 7.5	.05
Sports	46.7 ± 29.5	56.8 ± 25.0	<.01^a^	59.9 ± 27.0	67.0 ± 20.8	.22
QOL	63.4 ± 23.2	72.5 ± 19.8	<.01^a^	67.8 ± 23.6	70.4 ± 15.3	.59
KSS						
Objective knee score	19.0 ± 4.1	20.4 ± 3.2	<.01^a^	17.4 ± 6.4	19.6 ± 6.0	.13
Patient satisfaction score	27.2 ± 8.0	29.8 ± 8.0	.01^a^	24.0 ± 9.9	28.2 ± 10.7	.09
Patient expectation score	9.6 ± 2.8	10.3 ± 3.0	.05	10.6 ± 2.8	11.0 ± 3.0	.53
Functional knee score	66.8 ± 21.0	75.2 ± 16.6	<.01^a^	64.8 ± 21.6	75.7 ± 18.4	.03^a^

PROMs, patient-reported outcome measures; KSS, 2022 Knee Society Scoring System; ADL, activities of daily living; QOL, quality of life.

a *P* < .05.

## Discussion

The most important finding of the present study was the absence of differences in preoperative central sensitization and pain catastrophizing between patients who underwent UKA vs TKA. Although these psychological disorders had little effect on postoperative PROMs in the UKA group, they negatively affected postoperative PROMs in the TKA group. In other words, patients in the TKA group were more influenced by preoperative negative psychological status than those who underwent UKA. In clinical practice, CSI and PCS may be useful as a screening test for preoperative psychological intervention in especially TKA patients.

Although central sensitization and pain catastrophizing have become popular psychological assessments in knee arthroplasty, only one study has compared TKA vs UKA, and only in terms of pain catastrophizing. Birch et al. [16] compared preoperative pain catastrophizing between TKA and UKA using PCS and reported no difference in preoperative PCS scores between the procedures (*P* = .61). The PCS results are consistent with our results. Additionally, Wood et al. [22] reported that preoperative osteoarthritis grade did not influence preoperative pain catastrophizing status in patients who underwent TKA. Our preoperative knee range of motion in TKA was significantly lower than that in UKA, indicating more severe osteoarthritis in TKA; however, the grade of osteoarthritis did not influence PCS in our study. Whereas, preoperative PCS score before knee arthroplasty has wide variation among reports, with reported means between 12 and 25 points [22,[33], [34], [35]]. The wide range in PCS scores can be attributed to differences in patient characteristics and surgical indications across facilities, regions, and countries [34]. Lin et al. reported this variability in PCS scores among studies in their systematic review on PCS [34]. The proportion of patients with high PCS (PCS ≥30) was approximately 40% in both the UKA and TKA groups, and the PCS scores were relatively high in this patient cohort compared to other studies. Others have reported proportions of high PCS of 11.3% in UKA [36] and 15-27.5% in TKA [35,37,38]. However, in Japan, Hasegawa et al. [39] reported that 41% of patients score in the high PCS group before TKA, and their proportion of high PCS scores was almost the same as that in our study. When reviewing these studies, we could not identify specific reasons for the slightly higher proportion of patients with pain catastrophizing in our cohort; however, this could be related to cultural or epidemiological differences, because Japanese TKA patients have relatively high-grade osteoarthritis. Therefore, the differences in the PCS scores between the TKA and UKA groups should be evaluated in other regions or countries. Regarding central sensitization in knee arthroplasty, there have been no comparative subsidies between TKA and UKA; and, preoperative central sensitization before UKA in particular remains unclear. Our study reports the CSI in UKA for the first time, and the preoperative CSI in UKA was comparable with that in TKA. Several reports have reported preoperative CSI in TKA, with a preoperative average of 24–39 points and proportions of high CSI (CSI ≥40) of 24%–46% [10,27,28,40]. The CSI in this study was relatively lower; however, previous studies were mostly performed in South Korea. Therefore, regional characteristics may contribute to this difference, and further studies in other regions are needed.

Over the past decade, considerable attention has been focused on understanding the influence of psychological factors on TKA outcomes. To the best of our knowledge, this is the first cohort study to assess the relatively underexplored impact of pain catastrophizing and central sensitization on various patient-reported outcomes of both UKA and TKA. In this study, preoperative pain catastrophization and central sensitization negatively affected postoperative patient-reported pain and activity scores in TKA and had little effect on postoperative outcomes in UKA. Only one other study has investigated the influence of pain catastrophizing on outcomes following UKA. de Brauw et al. [36] reported that preoperative pain catastrophizing in patients undergoing UKA did not influence the EuroQol-5 Dimension–Visual Analog Scale and Forgotten Joint Scores, but negatively affected physical function in the KOOS at 1 year postoperatively. This partially aligns with findings of the present study, demonstrating that PCS itself did not correlate with any functional scores, but patients with high PCS scores (PCS>30) exhibited slightly lower functional scores, and there was no difference in pain scores on the KOOS and KSS compared with patients between high and normal PCS patients. Lin et al. systematically reviewed pain catastrophizing and PROMs in TKA and reported that some studies reported the association between the pain catastrophizing and postoperative pain score; however, 4 studies reported no association between pain catastrophizing and the postoperative pain score [34]. The investigators suggested that a multimodal investigation was necessary to explore the predictive fluctuations of pain catastrophizing on postoperative pain scores. To the best of our knowledge, no study has investigated the influence of central sensitization on outcomes after UKA. This study is the first to demonstrate that preoperative central sensitization has little effect on postoperative PROMs in UKA. However, there are several previous studies investigating preoperative central sensitization and postoperative PROMs in TKA [12,27,28,40], and their results are consistent with those of ours.

There appear to be 2 explanations as to why central sensitization and pain catastrophizing are negatively affected in TKA and have less influence on UKA. First is the preservation of native knee structures in the UKA, such as the anterior and posterior cruciate ligament, lateral compartment joint, and constitutional coronal alignment. Previous studies, which included both UKA and TKA, showed that UKA provides superior proprioception compared to TKA. Among TKA designs, cruciate-retaining TKA demonstrated better proprioception than posterior-stabilized TKA, likely due to preservation of the posterior cruciate ligament [41,42]. Additionally, compared with TKA, UKA has superior knee joint awareness (forgotten joint) [43] and better knee kinematics closer to that of the normal knee [44]. These advantages of UKA may not always be reflected in PROMs, and the difference may be small in aggregate analysis. However, patients with psychological problems could be sensitive to the discomfort caused by lower joint awareness and poorer kinematics in TKA. As a result, PROMs in patients undergoing TKA with psychological problems could be lower than in those without these disorders. In contrast, patients feel less discomfort after UKA than after TKA; therefore, PROMs in patients undergoing UKA could be less influenced by pain catastrophizing and central sensitization. Whereas, there is the second and alternative explanation that TKA patients often have worse preoperative PROMs and extensive surgery, therefore psychological factors might show up more in postoperative PROMs simply because there is more room for “dissatisfaction.” However, we do not have sufficient preoperative PROMs during this period; therefore, we will address this in a future study. Additionally, a greater invasiveness of TKA may increase the effect of preoperative psychological factors on postoperative PROMs comparing with a lower invasiveness of UKA.

The present study had several limitations. First, it was not randomized; as such, patient selection bias may have influenced the outcomes and confounding factors, such as smoking, alcohol, or pulmonary disease, may still remain in this nonrandomized study [45]. However, it is clinically impossible to randomly assign patients to TKA and UKA because the surgical indications differ for the 2 procedures. Second, this study was performed at a single institution, and the results may be inconsistent with those of other institutes. PCS and CSI scores vary widely across institutions, regions, and countries; therefore, multiple regional studies are under consideration. Third, preoperative demographics differed according to sex, height, and knee range of motion. However, we performed multivariable regression analysis to compensate for this issue. Fourth, although only a single implant was used for UKA, 3 different implants, which are primarily used at our institution, were included in the TKA group, as we aimed to capture comprehensive TKA patient data within our institutional preoperative psychological dataset. Postoperative PROMs differed slightly among the 3 implants; however, preoperative psychological factors did not differ among the groups (Supplementary Table 1). Further studies with larger cohorts including multiple implant types are needed to confirm these findings. Fifth, although the UKA group included 72 patients, which met the calculated minimum sample size, we acknowledge that the statistical power may have been insufficient to detect small effect sizes in the correlation analyses within this group. Therefore, the absence of significant associations in the UKA subgroup may reflect either a true lack of relationship or a type II error due to limited sample size. This should be considered when interpreting the subgroup results as well. Future studies with larger sample sizes are warranted to confirm these results. Sixth, the thresholds of CSI and PCS have not been established yet, therefore subgroup analysis between high and low CSI and PCS may not be proper and underpowered. However, these thresholds are most popular in available papers and these results are clinically valuable. Seventh, the 1-year follow-up period used in this study could be too short to evaluate long-term clinical outcomes and complications, though there is research indicating that PROMs after TKA show minimal change over a year [46]. Additionally, we could not calculate minimal clinically important differences for PROMs because preoperative PROMs were unavailable for our first several dozen cases. Eighth, this study contains osteoarthritis and osteonecrosis patients. However, there were no differences in CSI and PCS scores between the 2 groups and in PROMs between the 2 (Supplementary Table 2). Finally, larger prospective studies are needed because the present study may have been statistically underpowered, and there is ample potential for type II errors with regard to detecting all relevant outcomes. We are continuously collecting preoperative psychological data from patients undergoing knee arthroplasty, with the dataset now exceeding 1000 patients, and we are planning a worldwide psychological survey.

## Conclusions

Preoperative central sensitization and pain catastrophizing did not differ between patients undergoing UKA vs TKA. However, these psychological factors negatively affected postoperative PROMs in patients who underwent TKA, whereas, they were not found to significantly affect postoperative PROMs in the UKA group; however, this finding may reflect limited sample size rather than a true absence of effect. Clinically, there may be less need to account for preoperative psychological status in patients undergoing UKA; however, preoperative psychological status in those undergoing TKA is not negligible, and intervention would likely improve postoperative PROMs.

## Ethics statement

The institutional review board at the Tokyo University Hospital approved this retrospective study.

The patients and their families were informed that the patient data will be submitted for publication, and written informed consent was obtained from them.

## Data availability

The data used for this study are available on reasonable request in writing to the corresponding author.

## CRediT authorship contribution statement

**Kohei Kawaguchi:** Writing – original draft, Data curation, Conceptualization. **Ryota Yamagami:** Methodology, Investigation. **Kenichi Kono:** Methodology, Investigation, Data curation. **Shuji Taketomi:** Supervision. **Mei Lin Tay:** Writing – review & editing. **Simon W. Young:** Writing – review & editing, Supervision. **Hiroshi Inui:** Writing – review & editing, Methodology. **Sakae Tanaka:** Writing – review & editing, Supervision.

## Conflicts of interest

S.W. Young is on the speakers bureau/paid presentations for Stryker; has stock or stock options in Auckland Orthopaedics Ltd, Axis Sports Medicine, Surgical Solutions; and receives research support from Stryker and Smith & nephew; all other authors declare no potential conflicts of interest.

For full disclosure statements refer to https://doi.org/10.1016/j.artd.2026.102042.

## Figures and Tables

**Supplementary Table 1:** Preoperative psychological factors and postoperative patient-reported outcome measures (PROMs) among 3 total knee arthroplasty (TKA) prostheses

Items	TKA prosthesis			*P* value
	Journey	Sphere	Vanguard	
Knees	224	34	23	
CSI	22.0 ± 13.5	25.9 ± 15.5	23.4 ± 17.2	.44
PCS	25.7 ± 12.9	28.2 ± 13.5	28.2 ± 13.1	.32
KOOS				
Pain	88.2 ± 11.9^a^	77.3 ± 20.0^a^	75.3 ± 21.2^a^	<.01^a^
Symptoms	86.2 ± 11.1^a^	78.6 ± 14.1^a^	80.8 ± 16.5	<.01^a^
ADL	86.5 ± 12.1^a^	76.8 ± 18.1^a^	73.8 ± 18.4^a^	.01^a^
Sports	54.5 ± 27.4	42.3 ± 27.0	50.3 ± 22.4	.05
QOL	70.1 ± 21.3^a^	59.4 ± 23.0^a^	69.4 ± 21.5	.04^a^
KSS				
Objective knee score	20.2 ± 3.4^a^	19.0 ± 4.2	17.1 ± 4.1^a^	<.01^a^
Patient satisfaction score	29.8 ± 7.8^a^	25.5 ± 8.1^a^	24.2 ± 8.9^a^	.01^a^
Patient expectation score	10.1 ± 3.0	9.5 ± 2.7	10.4 ± 2.6	.50
Functional knee score	74.0 ± 18.1^a^	66.1 ± 19.0	58.6 ± 18.5^a^	<.01^a^

Average ±standard deviation.

CSI, central sensitization index; KSS, 2011 Knee Society Score; ADL, activities of daily living; QOL, quality of life; 95% CI, 95% confidence interval.

a *P* < .05.

**Supplementary Table 2:** Postoperative patient-reported outcome measures (PROMs) and demographic data between osteoarthritis and osteonecrosis knees in unicompartmental knee arthroplasty (UKA)

Items	Osteoarthritis	Osteonecrosis	*P* value
Knees	52	20	
Age (years old)	73.0 ± 11.1	72.3 ± 7.8	.79
BMI (kg/m2)	25.0 ± 3.8	25.0 ± 4.7	.96
CSI	22.5 ± 11.3	18.8 ± 8.0	.18
PCS	28.4 ± 10.8	24.4 ± 10.5	.16
KOOS			
Pain	89.8 ± 9.0	91.7 ± 12.1	.49
Symptoms	85.1 ± 11.1	88.3 ± 10.7	.31
ADL	85.6 ± 11.0	90.8 ± 8.3	.07
Sports	61.8 ± 24.1	68.3 ± 24.0	.32
QOL	66.6 ± 20.4	76.0 ± 15.8	.08
KSS			
Objective knee score	17.7 ± 6.4	20.5 ± 5.5	.09
Patient satisfaction score	25.3 ± 10.6	28.3 ± 10.0	.28
Patient expectation score	10.9 ± 2.7	10.6 ± 3.1	.70
Functional knee score	67.6 ± 19.8	76.2 ± 22.1	.12

Average ±standard deviation.

CSI, central sensitization index; KSS, 2011 Knee Society Score; ADL, activities of daily living; QOL, quality of life.

**STROBE Statement:** Checklist of items that should be included in reports of observational studies

	Item no.	Recommendation	Page No.	Relevant text from manuscript (line number)
Title and abstract	1	(*a*) Indicate the study’s design with a commonly used term in the title or the abstract	1	Line 1-2
		(*b*) Provide in the abstract an informative and balanced summary of what was done and what was found	1	Line 5-30
Introduction				
Background/rationale	2	Explain the scientific background and rationale for the investigation being reported	2	Line 37-66
Objectives	3	State specific objectives, including any prespecified hypotheses	3	Line 66-68
Methods				
Study design	4	Present key elements of study design early in the paper	5	Line 107-123
Setting	5	Describe the setting, locations, and relevant dates, including periods of recruitment, exposure, follow-up, and data collection	3-4	Line 72-85
Participants	6	(*a*) *Cohort study*—Give the eligibility criteria, and the sources and methods of selection of participants. Describe methods of follow-up *Case-control study*—Give the eligibility criteria, and the sources and methods of case ascertainment and control selection. Give the rationale for the choice of cases and controls *Cross-sectional study*—Give the eligibility criteria, and the sources and methods of selection of participants	3-4	Line 72-85
		(*b*) *Cohort study*—For matched studies, give matching criteria and number of exposed and unexposed *Case-control study*—For matched studies, give matching criteria and the number of controls per case		
Variables	7	Clearly define all outcomes, exposures, predictors, potential confounders, and effect modifiers. Give diagnostic criteria, if applicable	6	Line 125-132
Data sources/ measurement	8∗	For each variable of interest, give sources of data and details of methods of assessment (measurement). Describe comparability of assessment methods if there is more than one group	6-7	Line 134-146
Bias	9	Describe any efforts to address potential sources of bias	11	Line 256-258
Study size	10	Explain how the study size was arrived at	6-7	Line 134-146

Quantitative variables	11	Explain how quantitative variables were handled in the analyses. If applicable, describe which groupings were chosen and why	3-4	Line 72-85
Statistical methods	12	(*a*) Describe all statistical methods, including those used to control for confounding	6	Line 135-146
		(*b*) Describe any methods used to examine subgroups and interactions		
		(*c*) Explain how missing data were addressed		
		(*d*) *Cohort study*—If applicable, explain how loss to follow-up was addressed *Case-control study*—If applicable, explain how matching of cases and controls was addressed *Cross-sectional study*—If applicable, describe analytical methods taking account of sampling strategy	6	Line 135-146
		(*e*) Describe any sensitivity analyses		
Results				
Participants	13∗	(a) Report numbers of individuals at each stage of study—eg numbers potentially eligible, examined for eligibility, confirmed eligible, included in the study, completing follow-up, and analyzed	3-4	Line 72-80
		(b) Give reasons for nonparticipation at each stage		
		(c) Consider use of a flow diagram		
Descriptive data	14∗	(a) Give characteristics of study participants (eg, demographic, clinical, social) and information on exposures and potential confounders	7	Line 150-152
		(b) Indicate number of participants with missing data for each variable of interest		
		(c) *Cohort study*—Summarize follow-up time (eg, average and total amount)		
Outcome data	15∗	*Cohort study*—Report numbers of outcome events or summary measures over time	7	Line 152-155
		*Case-control study—*Report numbers in each exposure category, or summary measures of exposure		
		*Cross-sectional study—*Report numbers of outcome events or summary measures		
Main results	16	(*a*) Give unadjusted estimates and, if applicable, confounder-adjusted estimates and their precision (eg, 95% confidence interval). Make clear which confounders were adjusted for and why they were included	7-8	Line164-171
		(*b*) Report category boundaries when continuous variables were categorized		
		(*c*) If relevant, consider translating estimates of relative risk into absolute risk for a meaningful time period		

Other analyses	17	Report other analyses done—eg analyses of subgroups and interactions, and sensitivity analyses		
Discussion				
Key results	18	Summarize key results with reference to study objectives	8	Line 174-180
Limitations	19	Discuss limitations of the study, taking into account sources of potential bias or imprecision. Discuss both direction and magnitude of any potential bias	11-12	Line 256-283
Interpretation	20	Give a cautious overall interpretation of results considering objectives, limitations, multiplicity of analyses, results from similar studies, and other relevant evidence	8	Line 174-180
Generalizability	21	Discuss the generalizability (external validity) of the study results	8-9	Line 192-204
Other information				
Funding	22	Give the source of funding and the role of the funders for the present study and, if applicable, for the original study on which the present article is based	N/A	N/A

∗Give information separately for cases and controls in case-control studies and, if applicable, for exposed and unexposed groups in cohort and cross-sectional studies.

An Explanation and Elaboration article discusses each checklist item and gives methodological background and published examples of transparent reporting. The STROBE checklist is best used in conjunction with this article (freely available on the Web sites of PLoS Medicine at http://www.plosmedicine.org/, Annals of Internal Medicine at http://www.annals.org/, and Epidemiology at http://www.epidem.com/). Information on the STROBE Initiative is available at www.strobe-statement.org.

## Appendix A Supplementary data

Conflict of Interest Statement for Young

Conflict of Interest Statement for Kawaguchi

Conflict of Interest Statement for Inui

Conflict of Interest Statement for Kono

Conflict of Interest Statement for Tay

Conflict of Interest Statement for Taketomi

Conflict of Interest Statement for Tanaka

Conflict of Interest Statement for Yamagami
